# Supplementary material for: Ten-Eleven Translocation 1 and 2 Enzymes Affect Human Skin Fibroblasts in an Age-Related Manner
Source: Biomedicines. 2023 Jun 7;11(6):1659. doi: 10.3390/biomedicines11061659 (PMC10296407; doi:10.3390/biomedicines11061659)
Supplement: Supplementary file 1 [file biomedicines-11-01659-s001.zip › biomedicines-2282880-supplementary.pdf]

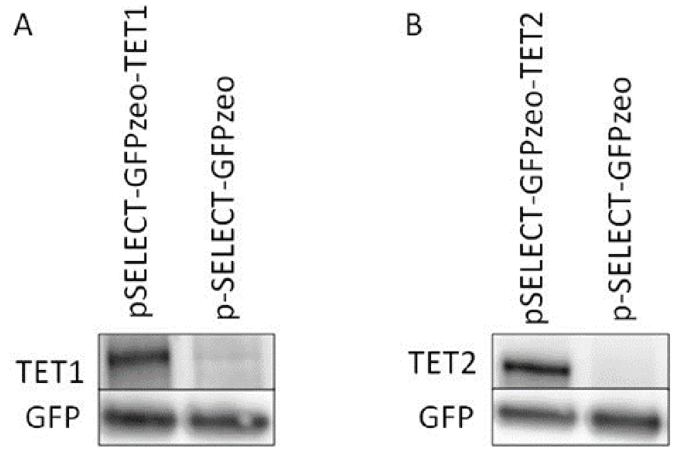

Figure S1. TET1 and TET2 expression in HEK293 cells transfected with pSELECT-GFPzeo-TET1 and pSELECT-GFPzeo-TET2 vectors. Cells transfected with “empty” pSELECT-GFPzeo vector served as a control. A. TET1. B. TET2.

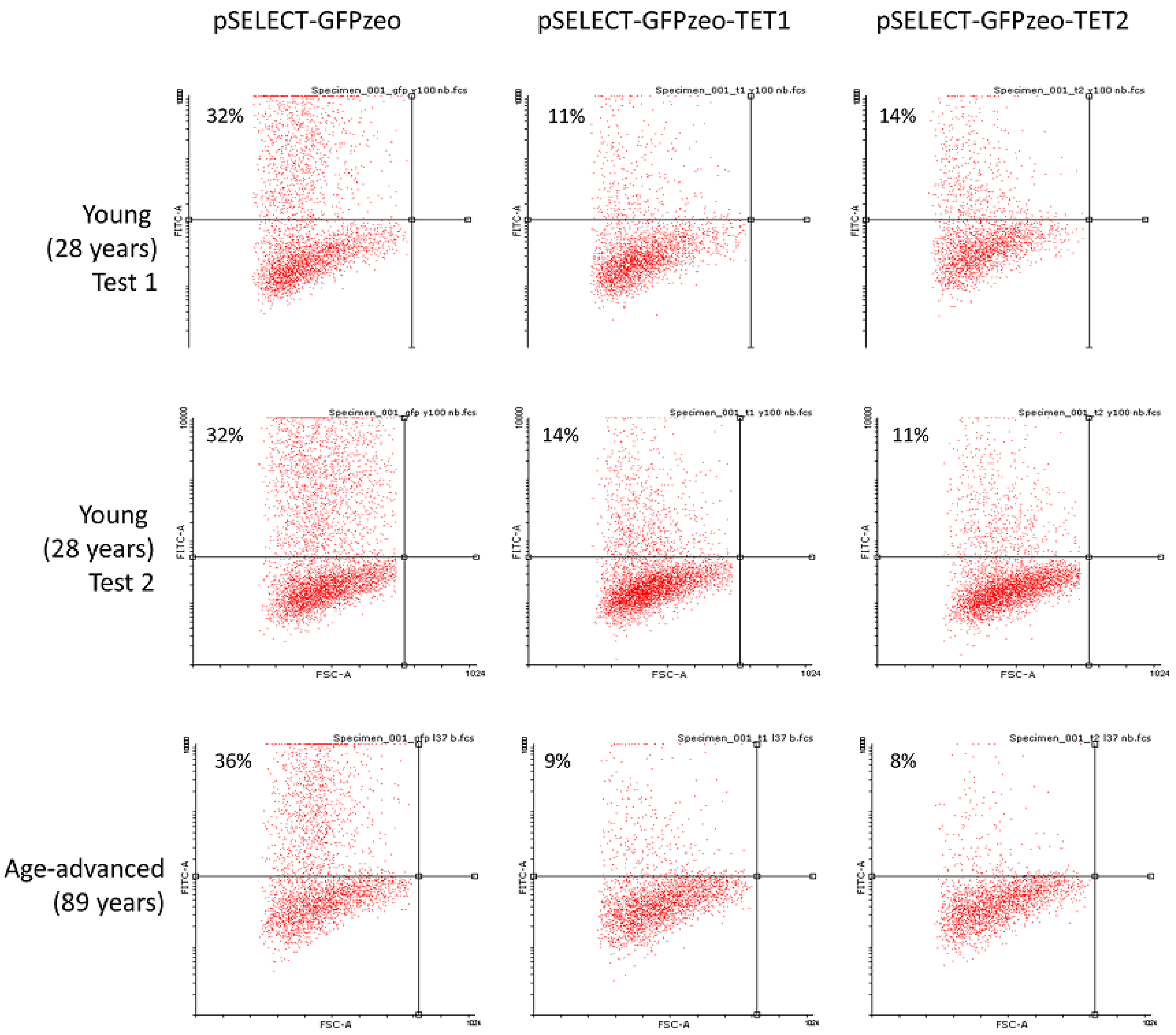

Figure S2. Representative flow cytometry plots showing the percentage of GFP-positive cells successfully transfected with pSELECT-GFPzeo control vector and pSELECT-GFPzeo-TET1 and pSELECT-GFPzeo-TET2 vector.
